# Antimicrobial and antioxidant activities of *Saccharomyces cerevisiae* IFST062013, a potential probiotic

**DOI:** 10.1186/s12906-017-1591-9

**Published:** 2017-01-21

**Authors:** Md. Fakruddin, Md. Nur Hossain, Monzur Morshed Ahmed

**Affiliations:** Industrial Microbiology Laboratory, Institute of Food Science and Technology (IFST), Bangladesh Council of Scientific and Industrial Research (BCSIR), Dhaka, Bangladesh

**Keywords:** *Saccharomyces*, Anti-bacterial, Probiotic, Anti-oxidant, Immuno, Activity

## Abstract

**Background:**

Probiotic yeast has become a field of interest to scientists in recent years.

**Methods:**

Conventional cultural method was employed to isolate and identify yeast and standard methods were used to determine different probiotic attributes, antimicrobial and antioxidant properties.

**Results:**

This study reports potential probiotic properties of a strain of *S. cerevisiae* IFST 062013 isolated from fruit. The isolate is tolerant to a wide range of temperature and pH, high concentration of bile salt and NaCl, gastric juice, intestinal environment, α-amylase, trypsin and lysozyme. It can produce organic acid and showed resistance against tetracycline, ampicillin, gentamycin, penicillin, polymixin B and nalidixic acid. It can assimilate cholesterol, can produce killer toxin, vitamin B12, glutathione, siderophore and strong biofilm. It showed moderate auto-aggregation ability and cell surface hydrophobicity. The isolate can produce enzymes such as amylase, protease, lipase, cellulose, but unable to produce galactosidase. The isolate can’t produce gelatinase and DNase. The isolate showed moderate anti-microbial activity against bacteria and fungi and cell lysate showed better antimicrobial activity than whole cell and culture supernatant. Again, the isolate showed better anti-bacterial activity against gram negative bacteria than gram positive. The isolate showed strong antioxidant activity, reducing power, nitric oxide and hydroxyl radical scavenging activity, significant brine shrimp cytotoxicity and acute toxicity and metal ion chelating activity. The isolate did not induce any detectable change in general health of mice upon oral toxicity testing and found to be safe in mouse model. The isolate improve lymphocyte proliferation and cytokine production in treated mice.

**Conclusions:**

Such isolate could be potential as probiotic to be used therapeutically.

## Background

Probiotics are a group of organism those confer health benefit to consumers [1]. To be used as probiotic, an organism should possess several attributes such as adhesive ability, acid and H_2_O_2_ production ability [2], bile tolerance and significant antibacterial activity and immunomodulatory activity [3] and must be non-pathogenic [4, 5]. Microorganisms that are probiotic to humans include yeasts, bacilli, *Escherichia coli*, enterococci, and the more commonly used bifidobacteria and lactic acid bacteria, such as lactobacilli, lactococci and streptococci [6]. Previous reports involving both In vitro and in vivo studies have indicated that *Saccharomyces boulardii* is able to prevent intestinal infection caused by *Escherichia coli*, *Salmonella typhimurium*, *Staphylococcus aureus*, *Pseudomonas aeruginosa, Proteus vulgaris*, *Yersinia enterocolitica* and *Candida albicans* [7]. But probiotics properties of *Saccharomyces cerevisiae* haven’t been explored that much.

*Saccharomyces cerevisiae* is a unicellular yeast and one of the most explored organism in terms of industrial applications and genetic studies [8]. Several previous studies showed that members of *Saccharomyces* genus can possess anti-bacterial and probiotic properties [9]. Several studies have also been reported with the use of yeasts (*S. boulardii or S. cerevisiae*) as a potential bio-therapeutic agent (probiotic) for the treatment of microbes associated diarrhea and colitis [10]. Anti-bacterial capability of *S. cerevisiae* might be due to production of extracellular protease [11], secretion of inhibitory proteins, stimulation of immunoglobulin A [12], acquisition and elimination of secreted toxins [13], killer toxins, sulfur di oxide etc. [14]. Foods such as milk, fermented foods, fruits, etc. are an important source of probiotic *Saccharomyces cerevisiae* [14, 15].

No such study has been performed in Bangladesh to assess the probiotic potential of indigenous *Saccharomyces cerevisiae*. This study aims to determine the probiotic properties of a putative probiotic yeast strain, *S. cerevisiae* IFST 062013.

## Methods

### Isolation and identification of *S. cerevisiae* IFST 062013

The yeast isolates, *S. cerevisiae* IFST 062013 was isolated from fruit and characterized morphologically and biochemically according to Fakruddin et al. [16]. Carbohydrate (Glucose, xylose, sucrose, fructose, galactose, lactose, maltose, trehalose, ribose, rhamnose, mannitol and dextrose) utilization capability of the isolate was determined according to Forouhandeh et al. [17]. Phylogenetic identification on the basis of sequencing of highly variable region of the fungal 5.8S rDNA gene was performed as described in Fakruddin et al. [16].

### Stress tolerance of yeast isolate

Sodium chloride tolerance of the yeast strains was performed according to Fakruddin et al. [18]. Sensitivity of yeast strains to oral and intestinal enzymes (lysozyme, trypsin and α-amylase) was studied as per Nowroozi et al. [19]. In vitro survival potential of the yeast isolates in simulated gastric environment (aqueous solution containing 3 g/l pepsin, and 5 g/l NaCl, pH 2.0) was determined according to Fietto et al. [20]. pH tolerance was determined according to Fakruddin et al. [21]. Bile salt tolerance of the isolates was investigated according to Kim et al. [22]. Thermotolerance of the yeast strains was determined according to Fakruddin et al. [14]. Organic acid production was determined according to Chowdhury et al. [4]. Antibiotic resistance of the isolate was determined by the standard agar disc diffusion technique described by Kirby-Bauer [23] and interpretation were taken from the CLSI standards [24].

### Probiotic properties

Cholesterol assimilation assay was performed as per Liong and Shah [25]. Cell surface hydrophobicity and auto-aggregation ability was performed according to Syal and Vohra [26]. Activities of enzymes (amylase, protease, lipase, galactosidase and cellulase) were determined according to Kim et al. [22] and production of gelatinase and DNase was determined according to Gupta and Malik [27]. Killer toxin production was observed according to Fakruddin et al. [16]. Vitamin B_12_ production by the isolate was assayed according to Bishnoi et al. [28]. The reduced glutathione (GSH) content in the yeast extracts and autolysates were determined according to Hassan [29]. Siderophore production was screened according to Sourabh et al. [15]. Biofilm formation assay was performed according to Li et al. [30].

### *Preparation of S. cerevisiae extracts* and autolysates

Yeast extracts from the yeast strains were prepared according to Ali et al. [31] and yeast autolysates were prepared according to Hassan [29].

### Antibacterial and anti-fungal activity

Anti-microbial (anti-bacterial and anti-fungal) activity of whole cell was performed by agar overlay method [32] and of cell culture supernatant and cell lysate was performed by well diffusion method [33]. Antibacterial activity was further characterized by determining whether bacteriostatic or bactericidal according to Chowdhury et al. [34]. All the test isolates of bacteria and fungi were taken from culture collection pool of Industrial Microbiology Laboratory, IFST, BCSIR, Dhaka.

### Antioxidant and toxicity properties

Total antioxidant capacity of yeast extracts and autolysates was assayed by the phosphomolybdenum method as described by Kumaran and Karunakaran [35]. The reducing power of yeast extracts and autolysates was determined by the method of Mathew and Abraham [36]. The antioxidant activity based on the scavenging activity of the stable DPPH free radical, was determined by the method described by Fakruddin et al. [37]. The scavenging activity of nitric oxide was determined by the method described by Kumaran and Karunakaran [35]. Hydroxyl radical scavenging activity was assayed by the method described by Nagai et al. [38]. Brine shrimp cytotoxicity assay was performed according to Fakruddin et al. [16] and acute toxicity was done according to Kabir et al. [39]. The ability of yeast extracts and autolysates to chelate ferrous ion was determined using the method described by Oboh et al. [40].

### Safety evaluation of *S. cerevisiae* IFST 062013

Twelve swiss albino mice aged 5–6 weeks were divided into two treatment groups designated as C and T (6 mice in each group). In order to assess the safety of the putative probiotic isolate, *S. cerevisiae* IFST 062013, a single dose of 150 μl (~10^9^ cfu) *S. cerevisiae* IFST 062013 were administered orally to each of the test group mice. Mice of the control group were fed with sterile PBS. After feeding, mice were monitored daily for 14 days to observe any changes in their activities, behavior and general health. Individual body weight was recorded daily using a balance [41]. In addition, the feces of mice were collected to enumerate the total numbers of *S. cerevisiae* and enterobacteria on day 0, 7 and 14. After 14 days. YPD agar was used for enumeration of *S. cerevisiae* and MacConkey agar was used for enumeration of enterobacteria [42]. Blood samples were collected for biomarker analysis, including aspartate aminotransferase (AST), alanine aminotransferase (ALT), alkaline phosphatase (ALP) and total cholesterol of the serum. Blood sample was also used to check fungaemia. The growth rate (GR), spleen weight index and liver weight ratio were calculated according to Kantachote et al. [43].

### Immuno-Modulatory activity of *S. cerevisiae* IFST 062013

Lymphocyte proliferation assay was performed according to Ren et al. [44]. Production of cytokines (IFN-α, IFN-γ, IL-10) was measured according to Ren et al. [44]. Gene expression of TLR-2, interferon (IFN)-γ, IL-4, Foxp3 and transforming growth factor (TGF)-β in intestinal mucosa was determined according to Zhu et al. [45].

### Statistical analysis

One way analysis of variance (ANOVA) was used to compare the results of the probiotic and control groups. Means, standard deviations and significant differences at *p* value < 0.05 were presented.

## Result

### Isolation and identification

Based on the colony characteristics (white and creamy texture) ovoid microscope shape, the presence of ascospore and budding pattern (multipolar), the selected isolate was found to belong S*accharomyces* type unicellular ascomycete. Ascospores formation by the yeast isolate was detected for indication of the ascomycetous yeast. The yeast isolate can produce pseudomycelium and showed in a filamentous form under microscope and can utilize glucose, fructose, sucrose, maltose and trehalose but failed to grow on lactose and xylose, rhamnose, raffinose and arabinose, which is characteristic of *Saccharomyces cerevisiae* [46]. 5.8 s rDNA sequencing revealed the identity of the isolate to be *Saccharomyces cerevisiae* (Accession no- HM134859.1).

### Stress tolerance

**Fig. 1:**
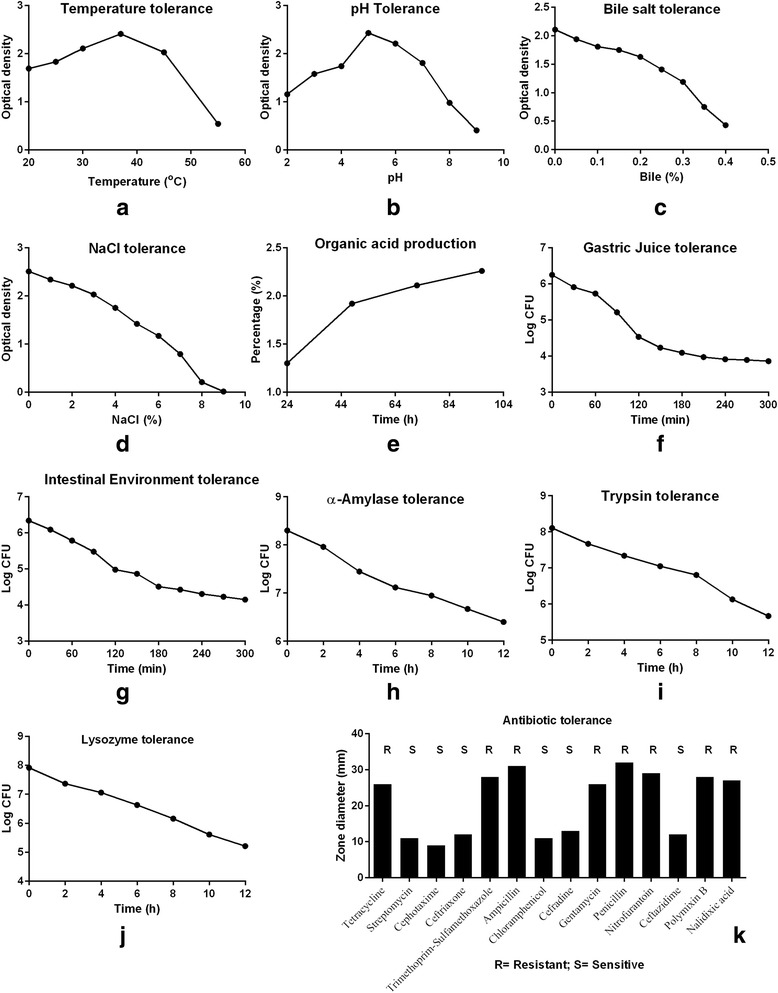
Stress tolerance of the *S. cerevisiae* IFST062013 isolate

Stress tolerance of the *S. cerevisiae* IFST 062013 isolate is shown in Fig. 1. The isolate able to survive in a wide range of temperature and pH with optimum conditions of 37 °C and pH 5.0. It can tolerate high concentration of bile salt and NaCl, gastric juice, intestinal environment, alpha-amylase, trypsin and lysozyme. It can also produce organic acid (2.25% after 90 h incubation). The isolate showed resistance to tetracycline, trimethoprim-sulphamethoxazole, ampicillin, gentamycin, penicillin, nitrofurantoin, polymixin B and nalidixic acid (Fig. 1).

### Probiotic properties

Probiotic properties of the isolate are shown in Table 1. The isolate can assimilate 33% cholesterol and produced different enzymes such as amylase (84 unit/g cell), protease (1760 unit/g cell), lipase (77 unit/g cell), cellulase (39 unit/g cell) and galactosidase as well as siderophore, killer toxin and strong biofilm. It can also produce 4.48 mg/100 ml total glutathione and 61.34% auto-aggregation ability (Table 1).

**Table 1:** Different probiotic properties of the yeast isolate

Property		*S. cerevisiae* isolate
Cholesterol assimilation		33%
Enzyme activity assay	amylase	84 unit/g cell
	protease	1760 unit/g cell
	lipase	77 unit/g cell
	cellulase	39 unit/g cell
*Killer toxin production*		+
total Glutathione production		1.48 mg/100 ml yeast
Galactosidase enzyme production		-
Production of siderophore		+
Biofilm formation		Strong (SBF > 1)
Auto-aggregation ability		61.34%

### Antibacterial and anti-fungal activity

Antibacterial activity of whole cells, culture supernatant and cell lysate of the isolated yeast is shown in Table 2. Comparing with doxycycline (30 μg/disc), the isolate showed moderate antibacterial activity. Antifungal activity of whole cells, culture supernatant and cell lysate of the isolated yeast is shown in Table 3. Comparing with fluconazole (100 μg/disc), the isolate showed moderate antifungal activity. In general, cell lysate showed better anti-bacterial and anti-fungal effect. Anti-bacterial effect of the isolate was better against gram negative pathogens than gram positive.

**Table 2:** Antibacterial activity of the yeast isolate

Test organism		Source ID (ATCC)	Zone diameter (mm)			
			Yeast isolate			Doxycycline
			Whole cell	Culture supernatant	Cell lysate	
Gram positive	*B. subtilis*	11774	7	5.1	11.6	21
	*S. aureus*	25923	7.5	4.9	10.3	24
	*B. cereus*	10876	7	6.1	9.4	12
	*B. polymyxa*	842	8.5	5.9	11.4	21
	*B. megaterium*	13578	7	5.4	9.8	28
	*E. faecalis*	29212	6.5	5.3	10.4	19
Gram negative	*S. typhi*	65154	11.5	8.3	14.8	27
	*S. flexneri*	12022	12.0	7.9	15.2	15
	*K. pneumoniae*	13883	10.5	7.5	13.7	22
	*P. vulgaris*	13315	10	8.1	15.1	17
	*E. coli*	25922	11	8.7	14.9	19
	*V. cholerae*	15748	13.5	9.6	16.3	26
	*P. aeruginosa*	27853	12.5	9.1	16.1	21

**Table 3:** In-vitro antifungal activity of CHET and fluconazole

Organism	Source ID (DSM)	Zone diameter (mm)			
		Yeast			Fluconazole
		Whole cell	Culture supernatant	Cell lysate	
*A. ustus*	63535	16.5	14.6	19.5	45
*A. niger*	737	27.2	24.3	33.4	65
*A. ochraceus*	824	21.6	18.4	24.1	41
*P. chrysogenum*	1075	23.5	19.7	25.3	48
*R. oryzae*	2200	19.7	17.5	22.7	46

### Antioxidant activity and toxicity

**Fig. 2:**
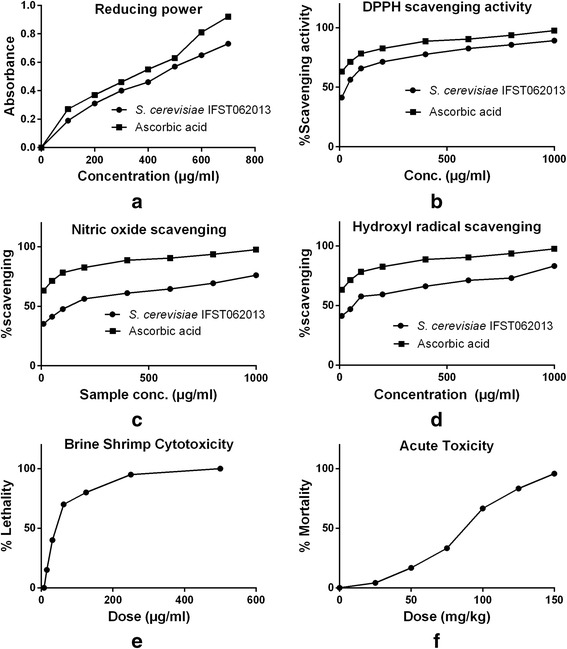
Pharmacological activity of the *S. cerevisiae* IFST062013 isolate

Antioxidant activities and toxic properties of the isolate is shown in Fig. 2. The isolate was found to possess different beneficial activity. The isolate showed significant reducing power, DPPH scavenging activity, nitric oxide scavenging and hydroxyl radical scavenging activity (comparing with ascorbic acid). Strong brine shrimp cytotoxicity and acute toxicity was shown by the isolate (100% lethality at 500 μg/ml in case of cytotoxicity and 150 mg/kg in case of acute toxicity). The chelating effect of the ferrous ions of the yeast isolate is presented in Table 4. The isolate exhibited the ability of iron binding.

**Table 4:** Ferrous iron chelation of yeast isolate and EDTA

SL	Sample concentration (mg/ml)	% Fe Chelation	
		Standard EDTA	Yeast isolate
1	2.5	47.86 ± 1.11	13.84 ± 0.97
2	3.5	63.24 ± 0.93	19.54 ± 1.15
3	4.5	76.15 ± 1.42	23.42 ± 0.68
4	5.5	86.34 ± 1.69	28.33 ± 1.43
5	6.5	91.25 ± 0.75	33.77 ± 1.85

Values are expressed as mean ± SD of three parallel measurements

### Safety evaluation of *S. cerevisiae* IFST 062013

**Fig. 3:**
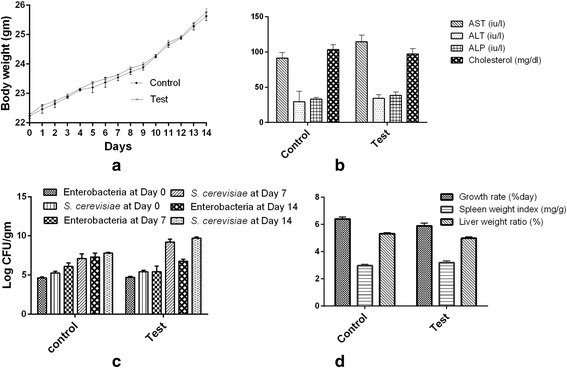
Safety evaluation of S. cerevisiae IFST 062013 in mice. **a** comparison of body weight; **b** comparison of AST, ALP, ALT and cholesterol level; **c** Enterobacteria and S. cerevisiae count in the feces; **d** comparison of liver weight and spleen weight ratio of treated and control mice

There were no significant differences in general health status between probiotic fed mice and control mice (Fig. 3a). No diarrheal death was observed and no *S. cerevisiae* detected in blood as well. Fungaemia was not observed in blood samples of the mouse. AST, ALT and ALP content in blood were similar and cholesterol content in treated mice blood is lower than the control mice (Fig. 3b). Enterobacteria and *S. cerevisiae* count in the feces of treated and control mice was almost similar during the observation period (Fig. 3c). The growth rate of treated mice found to be almost similar (difference non-significant) and the spleen weight index and liver weight ratio are almost similar in both groups (treated and control) (Fig. 3d).

### Immuno-modulatory activity

**Fig. 4:**
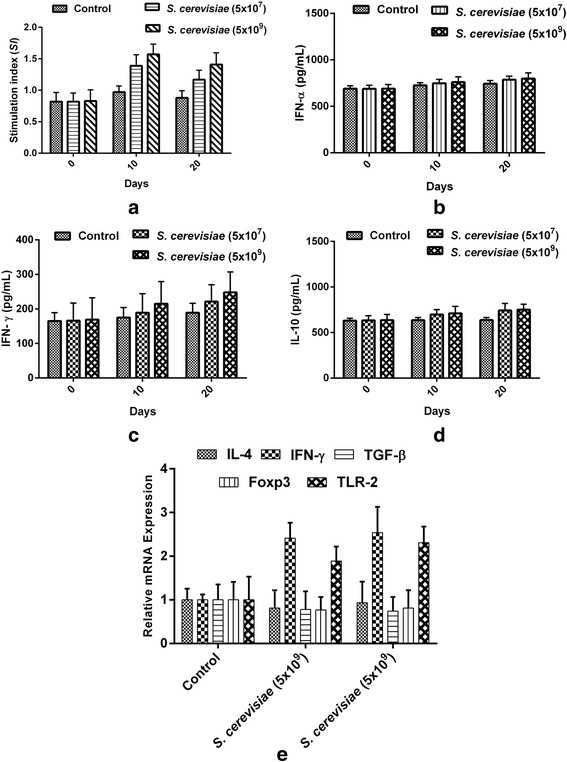
Immuno-modulatory activity of the S. cerevisiae IFST062013. **a** T-lymphocyte proliferation; **b** IFN-α, **c** IFN-γ, **d** IL-10 production in serum of treated and control mice; **e** Gene expression of cytokines (TLR-2, IFN-γ, IL-4, Foxp3 and TGF-β) in intestinal mucosa

Results indicate that the *S. cerevisiae* strain could stimulate a T-lymphocyte specific proliferative response. Proliferation index was significantly increased by the strains in a dose dependent manner (Fig. 4a). To evaluate the effects of *S. cerevisiae* IFST062013 on T-cell responses, the concentrations of IFN-α, IFN-γand IL-10 in mouse serum were examined. There was no significant difference in the induction of IFN-α production during the experimental period between treated and control group (Fig. 4b). IFN-γlevels in the serum showed no significant differences on day 10, but were, however, significantly increased by *S. cerevisiae* IFST062013 (248 pg/mL) at the higher dose (5x10^9^ CFU/mouse) compared with the control group (189 pg/mL) on day 20 (*P* < 0.05). IL-10 levels were significantly increased by *S. cerevisiae* IFST062013 (711 pg/mL) at the higher dose on day 10, compared with the control group (635 pg/mL) (*P* < 0.05), but a more prominent effect was found for probiotic treated group 751 pg/mL) compared with the control group (637 pg/mL) on day 20 (*P* < 0.01). Gene expression of cytokines (TLR-2, IFN-γ, IL-4, Foxp3 and TGF-β) in intestinal mucosa was determined. Expression of TLR-2 and IFN-γ was increased in mice treated the isolate in a dose dependent manner. In contrary, the expression of Foxp3, TGF-β and IL-4 was decreased (Fig. 4e).

## Discussion

*Saccharomyces cerevisiae* is one of the most studied microorganisms and for long has been used in different biotechnological applications due to its better fermentation capability. Besides industrial applications, probiotic and health benefit potential of yeast has also been reported in recent times [47]. Probiotics are defined as the viable microorganisms that exhibit a beneficial effect on health of the host by improving its intestinal microbial balance. *S. cerevisiae* and *S. boulardii* are clinically proven yeasts being used as a human probiotic and has shown to positively influence host’s health by antimicrobial effect, nutritional effect, inactivation of bacterial toxins, quorum sensing, trophic effects, immuno-modulatory effects, anti-inflammatory effects, cell restitution and maintenance of epithelial barrier integrity [48].

In this study a potential probiotic yeast strain (*S. cerevisiae* IFST 062013) was isolated from fruit and identified and characterized as *Saccharomyces cerevisiae* on the basis of morphological and biochemical characteristics and phylogenetic analysis. Many other studies reported probiotic yeast isolated from different samples [5, 14, 15, 49, 50]. Al Zubaidy and Khidhr [51] also identified *Saccharomyces cerevisiae var. bouldardii* from fruits with probiotic properties (antimicrobial activity, bile salt and gastric acid tolerance). Syal and Vohra [52] reported probiotic attributes of *Geotrichum klebahnii*, a yeast like fungus isolated from cheese.

To be a successful probiotic, any microorganisms must have the capability to be tolerant to stresses that prevail inside human body. The isolate can grow in a wide range of temperature and pH while optimum growth at 37^0^C and pH 5.0. It also possesses tolerance to bile salt, high NaCl, simulated gastric juice, intestinal environment, α-amylase, trypsin and lysozyme (Fig. 1). Syal and Vohra [26] reported yeast isolates that can survive in low pH and high bile salt concentration. It can produce organic acid and showed resistance against tetracycline, ampicillin, gentamycin, penicillin, polymixin B and nalidixic acid. The resistance of the yeast strain to antibiotics make it suitable for use in patients undergoing antibiotic treatment [52]. Higher resistance to antibiotic provides the yeast strain advantage over bacteria for therapeutic use.

The isolate pose desirable properties to be a potential probiotic. It can assimilate cholesterol (33%), can produce killer toxin, vitamin B12, glutathione, siderophore and strong biofilm. Vitamins play key role in numerous metabolic processes of the body and yeasts have been reported to be able to produce vitamins, especially vitamin B complex, which is a distinctive advantage for yeast to be used as a probiotic over bacteria [52]. Dubash et al. [53] reported a number of yeast strains belonging to *Sachharomyces cerevisiae, Candida pintolopesii, Candida tropicalis, Pichia anomala* and *Dekkera spp.* with killer toxin activity. It showed moderate auto-aggregation ability and cell surface hydrophobicity. Auto-aggregation and cell surface hydrophobility is very important property of a potential probiotic as these properties are involved in adhesion of the microorganisms to intestinal epithelial cells of patients [54]. To provide health benefits to patients by improving nutrient utilization within the intestine, a probiotic should have the ability to produce related enzymes [55]. The isolate can produce enzymes such as amylase, protease, lipase, cellulose, but unable to produce galactosidase. The isolate don’t produce gelatinase and DNase indicating its safety to be used for human patients as most of the pathogenic microorganisms produce these enzymes as part of their pathogenesis [26]. Cholesterol assimilation by yeast with probiotic attributes has also been reported by Chen et al. [53]. Syal and Vohra [26] reported yeast isolates that showed high auto-aggregation ability and cell surface hydrophobicity. The isolates were able to produce enzymes such as phytase, β-galactosidase, L-asparaginase, protease and lipase. The isolates can produce vitamin B12 and exopolysaccharide. The isolates can assimilate cholesterol, don’t produce DNase and gelatinase. Sourabh et al. [15] reported probiotic yeast with surface hydrophobicity and autoaggregation.

One of the most desirable properties of probiotic yeasts is the anti-bacterial activity of yeasts against human pathogens. The isolate showed moderate anti-microbial activity against bacteria and fungi in comparison with standard antibiotic (Doxycycline for bacteria and fluconazole for fungi). Cell lysate showed better antimicrobial activity than whole cell and culture supernatant. Again, the isolate showed better anti-bacterial activity against gram negative bacteria than gram positive. Culture supernatant showed least anti-microbial activity indicating that the anti-microbial compounds are not extracellular, rather cell bound. Rajkowska et al. [56] reported probiotic yeast strains (belonging to *S. cerevisiae* and *S. boulardii*) which showed antagonistic activity against human pathogens such as *Listeria monocytogenes, Salmonella typhimurium, Pseudomonas aeruginosa, Escherichia coli* and *Enterococcus faecalis*. Roostita et al. [14] reported yeast strains with antimicrobial activity against *Pseudomonas aerugenes*, *Staphylococcus aureus* and *Escherichia coli.* Syal and Vohra [26] isolated yeast with antimicrobial activity against *E. coli, Salmonella sp., Staphylococcus aureus, Vibrio cholerae* and *Pseudomonas* sp. Further studies on antimicrobial activity of the yeast isolate against other species of pathogenic bacteria and fungi are needed.

The isolate showed strong antioxidant activity, reducing power, nitric oxide and hydroxyl radical scavenging activity, significant brine shrimp cytotoxicity and acute toxicity (Fig. 2) and metal ion chelating activity (Table 4). Foligne et al. [42] reported yeast possessing significant anti-inflammatory activity in mice. Antioxidant activity of yeast has also been reported by Chen et al. [54]. Hassan [29] reported two yeast isolate, whose cell autolysates showed antioxidant and immunostimulating activity such as reducing power, DPPH radical scavenging, nitric oxide scavenging, hydroxyl radical scavenging and metal ion chelating activities. Sourabh et al. [15] reported probiotic yeast with antioxidant properties, DPPH free radical scavenging activity and siderophore production ability. The isolate also showed strong metal chelating activity, an essential property for antioxidant activity. Hassan [29] has reported probiotic *Saccharomyces cerevisiae* with strong metal ion chelating activity.

Safety assessment is an important criterion to select any potential probiotic for therapeutic applications. To assess the safety of *S. cerevisiae* IFST 062013, oral toxicity testing in mice was conducted. After 14 days of post-ingestions period, there were no significant differences in behavior or activity of the mice and no diarrheal death. No *S. cerevisiae* was detected in blood samples which indicate that the isolate don’t pose the ability to infiltrate areas outside the intestine. AST level provides a general estimation about any cellular injury occurred as its level increases in case of disease & cellular injury. On the other hand, ALT more specifically indicates liver cell damage & higher serum cholesterol. Increased ALP has been linked with increased osteoblastic activity & lack of bile flow & higher serum cholesterol [41]. Blood sample analysis also showed that AST, ALP and ALT content is almost similar in both treated and control group mice. But cholesterol content in treated group mice were lower than control group mice further ensuring the isolate’s ability to assimilate cholesterol. These observations indicate that the isolate do not induce any gross acute oral toxicity on general health, growth and development of mice. There were no significant differences in numbers of enterobacteria and *S. cerevisiae* in the feces of treated group and control group mice throughout the 14 day observation period, which indicate that the isolate can persist in the intestines. Growth rate of the treated group mice was almost similar to that of the control mice. There were no significant difference between spleen weight index and liver weight ratio of the treated group and control group mice. These results indicate that the isolate cannot induce any systemic infections in mice and is non-invasive.

To test the effect of *S. cerevisiae* IFST062013 on the cellular immune response, we examined splenocyte proliferation. On day 10, the spleen lymphocyte proliferation capacity was significantly increased in the *S. cerevisiae*-treated groups when compared with the ConA control group (*P* < 0.04). The SI values of the higher dose groups (5x10^9^ CFU/mouse) reached their maximum values and were higher than for the moderate dose groups (5x10^8^ CFU/mouse) (*P* < 0.01). On day 20, the results showed a similar trend. These results indicate that the probiotic *S. cerevisiae* strain could stimulate a T-lymphocyte specific proliferative response and could potentiate humoral immunity and cell-mediated immunity and consequently have potential antitumor activity. Cytokines play an important role in the development of immune response, we evaluated the effect of the strain on the production of pro-inflammatory cytokines IFN-α and IFN-γ, and the anti-inflammatory cytokine IL-10. IFN-γinduces cell-mediated and inflammatory immune responses. Our results showed that the probiotic strain simultaneously induced pro- and anti-inflammatory mediators and consequently helped to maintain a balance between Th1 and Th2 type cytokines, which is important for host immunity. The probiotic strain modulates gene expression of cytokines in dose dependent-manner (Fig. 4).

## Conclusion

The *Saccharomyces cerevisiae* IFST 062013 isolate showed promising probiotic activities and possessed comparable attributes with other reported probiotic yeasts. Continuous screening for selection of probiotic strains with even better attributes should be carried out. Before therapeutic application, further research should be done to ensure safety and efficiency of the potential probiotic yeast.

## Abbreviation

ALP: Alkaline phosphatase
ALT: Alanine aminotransferase
AST: Aspartate aminotransferase
Cfu: Colony forming unit
CLSI: Clinical and Laboratory Standards Institute
DNA: Deoxyribo nucleic acid
DPPH: 2,2-diphenyl-1-picrylhydrazyl
GSH: Glutathione
PBS: Phosphate buffered saline
PCR: Polymerase chain reaction
YPD: Yeast extract-Peptone-Dextrose agar
µg: Micro gram
